# Structure of Ljungan virus provides insight into genome packaging of this picornavirus

**DOI:** 10.1038/ncomms9316

**Published:** 2015-10-08

**Authors:** Ling Zhu, Xiangxi Wang, Jingshan Ren, Claudine Porta, Hannah Wenham, Jens-Ola Ekström, Anusha Panjwani, Nick J. Knowles, Abhay Kotecha, C. Alistair Siebert, A. Michael Lindberg, Elizabeth E. Fry, Zihe Rao, Tobias J. Tuthill, David I. Stuart

**Affiliations:** 1 Division of Structural Biology, University of Oxford, The Henry Wellcome Building for Genomic Medicine, Headington, Oxford OX3 7BN, UK; 2 The Pirbright Institute, Pirbright, Surrey GU24 0NF, UK; 3 National Laboratory of Macromolecules, Institute of Biophysics, Chinese Academy of Science, Beijing 100101, China; 4 Department of Chemistry and Biomedical Sciences, Linnaeus University, SE-391 82 Kalmar, Sweden; 5 Laboratory of Structural Biology, School of Medicine, Tsinghua University, Beijing 100084, China; 6 Diamond Light Sources, Harwell Science and Innovation Campus, Didcot OX11 0DE, UK

## Abstract

Picornaviruses are responsible for a range of human and animal diseases, but how their RNA genome is packaged remains poorly understood. A particularly poorly studied group within this family are those that lack the internal coat protein, VP4. Here we report the atomic structure of one such virus, Ljungan virus, the type member of the genus *Parechovirus* B, which has been linked to diabetes and myocarditis in humans. The 3.78-Å resolution cryo-electron microscopy structure shows remarkable features, including an extended VP1 C terminus, forming a major protuberance on the outer surface of the virus, and a basic motif at the N terminus of VP3, binding to which orders some 12% of the viral genome. This apparently charge-driven RNA attachment suggests that this branch of the picornaviruses uses a different mechanism of genome encapsidation, perhaps explored early in the evolution of picornaviruses.

## Abstract (web summary)

The Ljungan virus is a picornavirus that lacks the internal coat protein VP4, and the packaging of its RNA genome is poorly understood. Here, the authors use cryo-electron microscopy to visualize this virus and suggest that it uses a different mechanism to other viruses for encapsidation of its genome.

## Graphical abstract

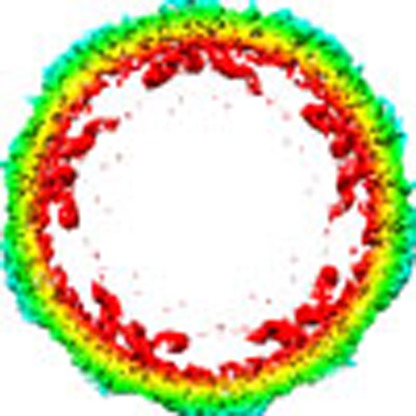

Encapsidation of the viral genome leading to the assembly of infectious progeny virions is an essential step in the virus life cycle. The most commonly employed strategies for packaging viral genomes involve either filling a preformed procapsid with progeny genome or nucleation of capsid assembly around viral nucleic acids^1^,^2^. We have a convincing picture of how some bacteriophages package the viral genome into a preassembled procapsid using packaging motor proteins^3^, and how plant viruses govern RNA packaging through protein–protein or protein–RNA interactions^4^; however, picornavirus assembly remains much less clear. Picornaviruses, small (300 Å diameter) single-stranded, positive-sense, RNA viruses, with a genome of between 7.1 and 9.7 kb, are responsible for a variety of human and animal diseases and include poliovirus (PV), human rhinovirus, enterovirus A71, hepatitis A virus (HAV) and foot-and-mouth disease virus^5^. The selective encapsidation of the viral genome, and not cellular RNAs, requires the specific recognition of packaging signals—sequences or structures unique to viral nucleic acid. Numerous attempts to identify an RNA encapsidation signal in enterovirus genomes have failed^6^, although a recent study reported that PV (*Enterovirus* genus) encapsidation is facilitated by the interactions of capsid proteins and the RNA replication complex^7^ and Aichi virus (*Kobuvirus* genus) was reported to contain a 5′-terminal RNA stem–loop critical for viral RNA encapsidation^8^. However, none of these studies have provided structural evidence to explain encapsidation.

Ljungan virus (LV), a picornavirus originally isolated from voles, has been proposed as a zoonotic virus, potentially associated with type-1 diabetes mellitus, myocarditis and Guillain–Barré syndrome in humans^9^. Currently, four LV genotypes have been characterized (LV-1 to LV-4), two isolated in Sweden and another two in the United States^10^. LV belongs to the *Parechovirus* genus and is closely related to human parechoviruses (HPeV) (∼48% amino-acid identity between the VP0 proteins)^11^,^12^. Parechoviruses exhibit several distinctive features, including the lack of the final, assumed RNA-catalysed, maturation cleavage of the capsid protein precursor VP0 to VP2 and VP4 (refs 12, 13, 14, 15), the existence of an ∼20-amino-acid extension enriched with basic residues to the N terminus of VP3 (ref. 15) and distinctive 2A proteins with unknown function^16^. The lack of VP4 in these viruses, which is implicated in forming membrane pores crucial for viral uncoating^17^, suggests that the parechoviruses may uncoat via a different mechanism. Compared with HPeV, the N terminus of LV VP0 is further shortened by some 30 residues and does not contain the myristylation signal GXXX(S/T) typical of the majority of picornaviruses^18^. A further major difference between HPeV and LV is located at the C terminus of VP1—LV does not possess the arginine–glycine–aspartic acid (RGD) motif important for cell surface interaction with integrin receptors in most HPeVs^19^,^20^, but instead contains a unique 42-amino-acid extension. To date, no receptors have been identified for LV.

For many picornaviruses, two predominant types of viral particles are produced during a natural infection, mature virus (containing packaged RNA) and empty procapsids (without RNA), which may be separated using continuous sucrose density gradient ultracentrifugation^21^. However, such natural empty particles have not been observed for either LV or HPeV^22^. The reasons for the absence of empty particles are unclear, but may reflect a distinct assembly mechanism. It is conceivable that the basic N-terminal A/LRM (arginine/lysine-rich motif) of VP3 might be involved in RNA–protein interactions and this, together with the fact that viral RNA does not cleave VP0 into VP2 and VP4, could reflect an altered mechanism of genome encapsidation for parechoviruses. Assembled LV particles are robust, so that even heat treatment, which tends to produce modified enterovirus particles^23^, does not produce detectable alteration in LV particles^22^. Furthermore, LV particles are reported to be resistant to acidic pH, detergents and oxidizing environments with complete inactivation of crude extracts requiring heating to 90 °C (ref. 24).

Although an 8.5-Å cryo-electron microscopy (cryo-EM) structure of HPeV-1 has been reported^15^, there is no high-resolution structural information available for parechoviruses. Here we report the determination of an atomic model of LV from a 3.78-Å resolution cryo-EM analysis. Structural comparisons place the virus close to HAV within the evolutionary hierarchy of picornaviruses, while substantial differences on both the inner and outer capsid surfaces, including the visualization of 12% of the RNA genome forming icosahedrally ordered interactions with the capsid, suggest the use of a charge-driven mechanism for genome encapsidation.

## Results

### Characterization and structure determination

An infectious complementary DNA (cDNA) clone was previously constructed from the cytolytically replicating cell-culture-adapted LV variant 87-012G^25^. Virus generated from this cDNA clone produced a clear cytopathic effect (CPE) at 3 days post infection. LV was grown in GMK cells and purified by centrifugation, linear and discontinuous sucrose gradient ultracentrifugation and ultrafiltration (See Methods). Unlike other picornaviruses, for example, enterovirus A71, CVA16 and HAV, only one band was observed following rate-zonal centrifugation and characterized by SDS–polyacrylamide gel electrophoresis (Supplementary Fig. 1a,b). The PaSTRy assay^26^, *λ*260/*λ*280 absorbance ratios (1.64) and negative-stain transmission electron microscopy (Supplementary Fig. 1c) indicated that this band comprised mature virions, containing RNA.

**Figure 1:**
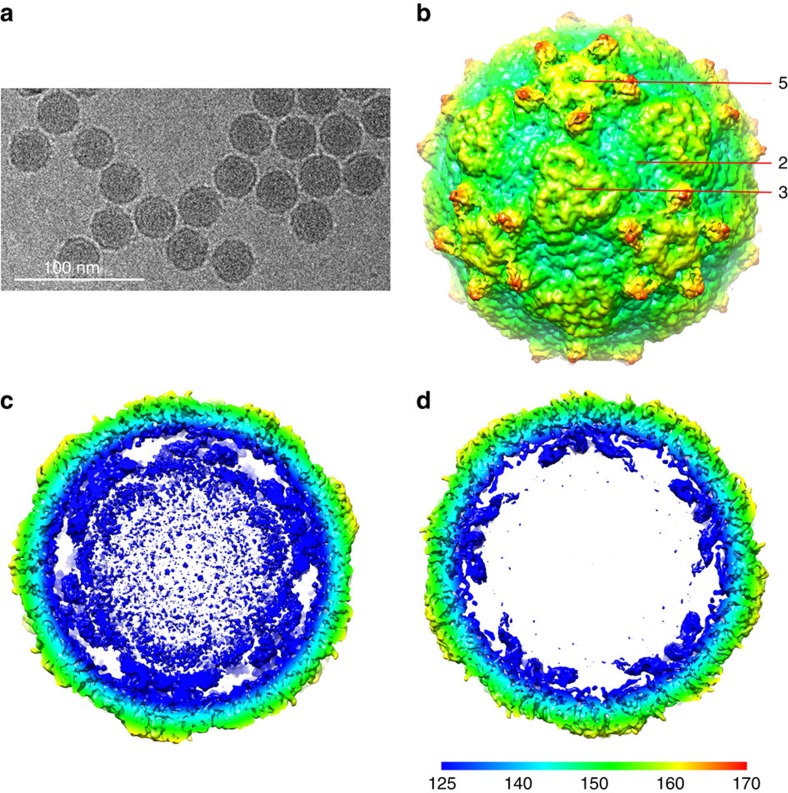
Single-particle 3D reconstructions of LV. (**a**) Cryo-EM image of LV. (**b**) 3D reconstruction of LV particle viewed down the three-fold axis. The surface is coloured by radius from blue through green to red from the lowest to the highest radius. The icosahedral five-fold, three-fold and two-fold axes are labelled as 5, 3, and 2, respectively. (**c**) A thin slice of the central section of the LV particle viewed down the three-fold axis. (**d**) The central section of the LV reconstruction rendered at higher contour level (4σ above the mean), viewed along a five-fold axis of symmetry and depth queued to reveal the five high-density protrusions inside the capsid under each vertex.

**Figure 2:**
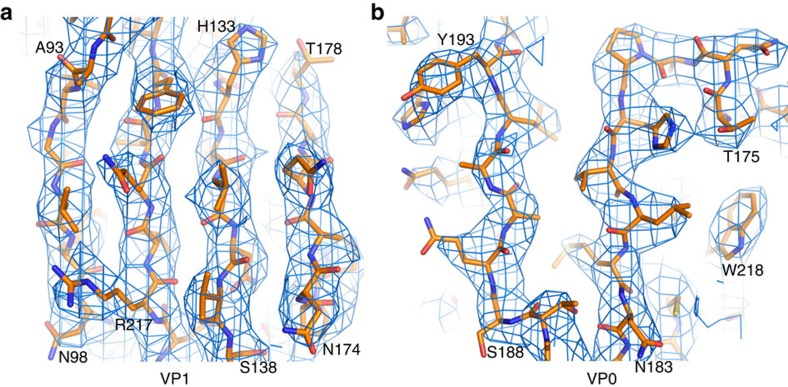
Electron density maps. (**a**) From a section of VP1. (**b**) From a section of VP0.

Cryo-EM micrographs of purified LV virions were recorded using a FEI Polara electron microscope equipped with a Gatan K2 Summit detector (see Methods). A total of 5,558 virions were selected from the cryo-EM images and subjected to two-dimensional (2D) alignment and three-dimensional (3D) reconstruction by single-particle techniques (Fig. 1) using EMAN2 and Relion^27^,^28^. Image processing and 3D reconstruction adopted truly independent refinements^29^ and the resolution was assessed using the ‘gold' standard Fourier shell correlation (FSC)=0.143 criterion^30^,^31^. The final resolution was 3.78 Å (Table 1; Supplementary Fig. 2). The polypeptide main chain and side chains were well resolved for most of the capsid (Fig. 2), allowing an atomic model for the majority of the three capsid proteins to be manually built in. The model was refined^32^ using standard X-ray crystallographic metrics, including *R*-factor, real-space correlation and Ramachandran plots, to validate the refined structure (Table 2).

### Unusual surface features

**Figure 3:**
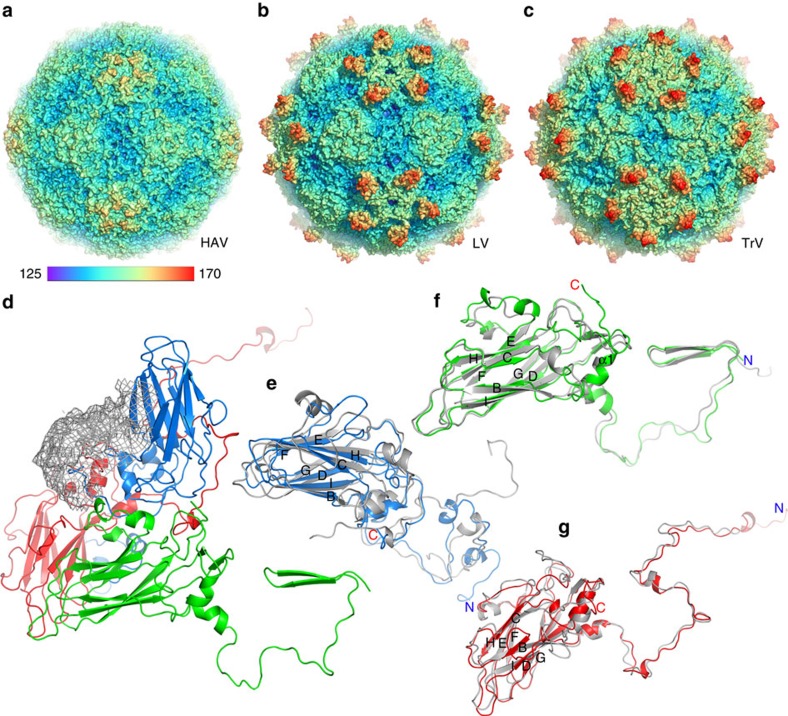
Overall structure of LV. (**a**–**c**) Comparison of capsids from LV (**b**) with HAV (**a**) and TrV (**c**). The capsids are coloured by radius from blue to red according to the scale bar shown. (**d**) Ribbon diagram of the LV protomer. VP1, VP0 and VP3 are shown in blue, green and red, respectively; the VP1 C-terminal 55 residues that form the surface protrusion around the five-fold axis are shown by a lower-resolution electron density map. (**e**–**g**) Ribbon diagrams showing the LV capsid proteins VP1 (**e**), VP0 (**f**) and VP3 (**g**) with the individual strands and termini labelled, compared with those of HAV (grey), the N- and C termini of the LV proteins are labelled in blue and red, respectively.

The LV capsid proteins are mostly well ordered, apart from residues 1–30 of VP1, 1–36 of VP0 and 1–3 of VP3. There are very marked (∼20 Å high) protrusions displaced some 45 Å from the icosahedral five-fold axes, but otherwise the external surface is broadly similar to that of other picornaviruses; being relatively smooth it is most similar to HAV^21^, although less angular (Fig. 3a,b). There is no trace of the enterovirus canyon, often the site of receptor binding^33^, since shortened VP1 and VP0 loops ablate the north and south walls of the canyon, in a very similar fashion to HAV. The transverse central sections of the LV density maps (Fig. 1c,d) emphasize the internal differences, revealing genomic RNA involved in visible contacts with the protein shell at the five-fold axes.

### Stability of LV

LV capsids from crude extracts have been reported to be able to withstand high temperature and low pH (retaining infectivity above 70 °C and at pHs down to about 4), which would make them equally robust with HAV capsids^21^. However, our results (Supplementary Fig. 3) show that genomic RNA becomes exposed at a significantly lower temperature (∼60 °C) with little variation in stability with pH, this being ∼10 °C lower than the capsid dissociation temperature of HAV. In fact, this is broadly in line with previous experiments with purified LV^24^, which suggests that an exogenous factor may stabilize the particles in infected cells. In enteroviruses, capsid protein melting occurs in two distinct steps indicative of a two-stage transition in protein conformation, whereas LV only undergoes the higher-temperature conformational transition, consistent with the observation that alternative particles of parechoviruses cannot be produced by heat treatment^22^. These results, along with the lack of VP4, indicate that LV probably uncoats via a novel mechanism, which is different from those utilized by most other picornaviruses^21^. In line with this, monitoring of LV replication by immunofluorescence staining suggested direct cell-to-cell transmission^25^, which was also proposed for HAV^21^.

### The organization of VP1-3 resembles that observed in HAV

The capsid proteins (VP0, VP1 and VP3) adopt the expected eight-stranded anti-parallel β-barrel configuration and are arranged with pseudo *T*=3 symmetry (where *T* is the triangulation number), such that VP1 surrounds the five-fold axes and VP0 and VP3 alternate about the two- and three-fold axes. Structure-based sequence alignment allows us to make a robust sequence alignment revealing limited similarity (∼25% identity) to other picornaviruses (Supplementary Fig. 4). Overall, HAV has the most similar known structure to LV and the fold of the three proteins in the two viruses are compared in Fig. 3d–g. Perhaps correlated with the absence of a canyon, LV does not harbour a continuous hydrophobic pocket in VP1 (Supplementary Fig. 5a), such as that seen in enteroviruses^34^ where expulsion of a lipidic pocket factor is a pre-requisite for uncoating^35^. LV VP1 most closely resembles that of HAV, which likewise does not have a pocket factor (root mean squared deviation for 83% of Cαs is 2.0 Å); similar characteristics between the two viruses in VP1 are: a lengthened βH strand and alternative conformation of the GH loop blocking the entrance to the pocket, while the βC, βE, βF and βH strands shift so that it can no longer accommodate a lipidic pocket factor. There are nonetheless differences in the conformation of the CD, EF and GH loops between the LV and HAV VP1 structures (Fig. 3e). The LV GH loop is three residues shorter (one of the shortest in any picornavirus) and the CD and EF loops are nine and seven residues shorter, respectively. The major difference between LV and HAV VP1, however, occurs at the C terminus, owing to the ∼50 residue extension in LV (Supplementary Fig. 4 and next section). LV VP0 is strongly similar to HAV VP2 (root mean squared deviation for 206 Cαs (95%) is 1.6 Å). Sequence alignment indicates a short counterpart (∼27 residues) of HAV VP4 in LV VP0 (Supplementary Fig. 4), although there is no reported cleavage of VP0 into VP2 and VP4 in the genus *Parechovirus*^14^. Both HAV VP4 and the LV VP0 N terminus (residues 1–36) are disordered. The N terminus of HPeV VP0 is extended by some 30 residues compared with LV. LV uses the VP2 N-terminal domain swap across the icosahedral two-fold axis, first observed in a picornavirus for HAV^21^ (and seen in the insect picorna-like viruses^36^), possibly imparting extra stability at this interface (Fig. 3d,f). This arrangement is presumably common to all members of the genus. Furthermore, the interaction region (VP2 αA helix) adjacent to the icosahedral two-fold axes, which separates during the initial stages of enterovirus uncoating^35^,^37^, shows tight packing in LV (3.7 Å separation), as was seen in HAV^21^, possibly further contributing to the stability of the LV particles. The overall fold of VP3 is similar to other picornaviruses; however, LV and parechoviruses have an N-terminal extension, which is responsible for interacting with RNA (see below). LV VP3 has a puff structure connecting the αA helix and βB strand, which is absent in HAV, but the latter has an additional puff structure between the βC strand and αB helix of VP3 (Supplementary Fig. 4). There are also differences between the two viruses in the conformation of the VP3 GH loop (Fig. 3). We would expect strong similarity between the structure of LV and HPeVs; however, antibodies raised against the VP0 and VP1 capsid proteins of LV, while recognizing the LV virion, do not recognize HPeV-1 by immunofluorescence of infected cells^38^.

### Surface projections surround the five-fold axes

The external surface of the LV capsid possesses unusual protrusions surrounding the five-fold axes, which extend the radius of the LV particle to ∼170 Å (Fig. 3b). Although these are poorly ordered and best seen in lower-resolution reconstructions, it is unambiguous that they are formed from the C terminus of VP1. The disorder precludes tracing the protein backbone for this domain (residues 242–297) but the predicted protein secondary structure would suggest three helices. Such a protrusion formed from a C-terminal extension is unique in picornaviruses; however, in Seneca valley virus, there is a similarly sized and placed projection formed by loops of VP1 and VP2, and in the insect picorna-like virus Triatoma virus (TrV^39^) there are such projections formed from insertions within the loops and β-sheets of VP1 and VP3, although displaced by ∼13 Å (Fig. 3). The 55 amino acids contributing to this structure have a high proportion of charged residues (13 negatively and 8 positively charged residues). Some other picornaviruses, for example, CV-A9 and HPeV-1, possess an arginine–glycine–aspartic acid (RGD) motif at the C terminus of VP1 involved in integrin recognition; however, this motif is not present in the LV VP1 extension, which appears to contain no distinctive sequence motifs (Supplementary Fig. 4), although it shares 40% identity with the universal stress protein of *Streptomyces auratus*. Cell-culture-adapted virus able to replicate more efficiently has been found to have three mutations in this region: Y289H in VP1 and A162T and S172G in VP0 (ref. 40) (Supplementary Fig. 5b). The VP0 mutations are located at either end of the EF loop and while not surface exposed, they may well alter the surface conformation, especially since A162T is close to a disulphide bond between Cys 191 (GH loop) and Cys 124 (CD loop) of VP1. Y289H is located in the VP1 C-terminal extension (Supplementary Fig. 5b), disordered density for which is located above the junction of the three capsid proteins within a biological protomer (Fig. 3d) and is therefore adjacent to the VP0 EF loop. LV encodes two 2A proteins, 2A1 and 2A2. The 20-residue-long aphthovirus-like 2A1 protein possesses a DvExNPGP motif proposed to mediate cleavage of the LV polyprotein *in vitro* and may constitute the carboxy terminus of the structural protein VP1 (ref. 13). It is not yet resolved as to whether this forms part of the extra density we see in the five-fold protrusion. However, in HAV protein VPX (VP1+2A) is present during particle assembly and the 2A protein is believed to have a role in directing assembly, before being cleaved off. It is possible that the 2A1 of LV also has a role in particle assembly, and may likewise be cleaved on particle maturation.

### The N-terminal region of VP3 binds an RNA stem–loop

**Figure 4:**
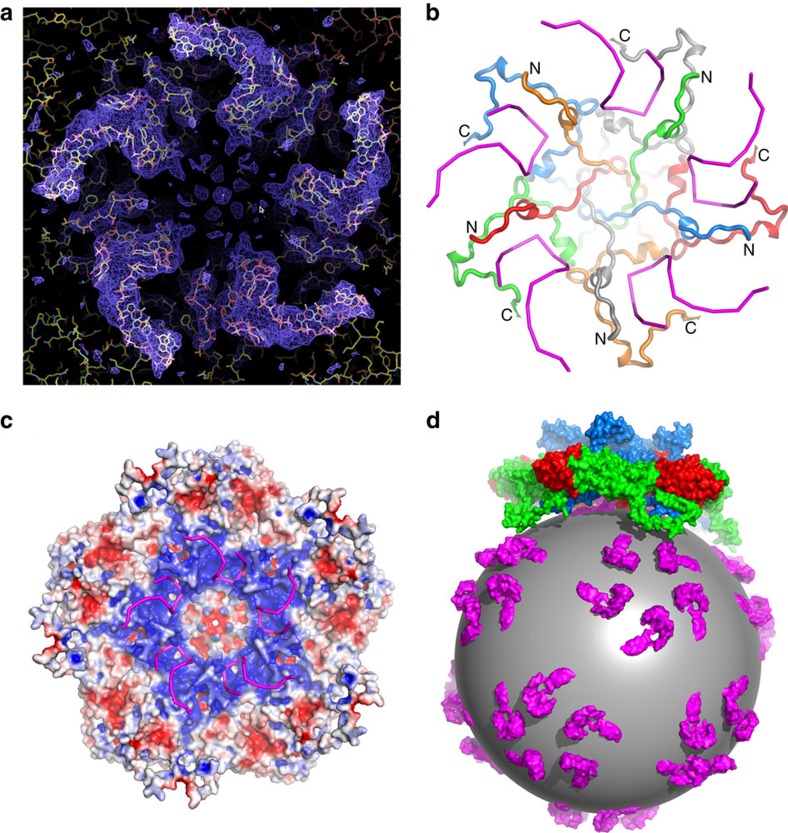
The partial structure of the LV genome and virus encapsidation. (**a**) Electron density for the N termini of VP3 and the ordered RNA around the five-fold axis. (**b**) The structure of VP3 N termini around the five-fold axis (individual copies distinguished as blue, green, red, orange and grey ribbons, from residue 4 labelled N to residue 64 labelled C) and their proximity to the RNA (magenta). (**c**) Inner electrostatic surface of the LV pentamer showing the predominance of positive charge that interacts with the backbone of the genomic RNA (magenta sticks). (**d**) Cartoon diagram representing the LV genomic RNA as a sphere with ordered fragments forming pentameric protrusions (magenta); the pentamers of capsid protein associate with the genome RNA through electrostatic interaction.

The extended N-terminal region of VP3 was clearly defined in the density map and was modelled starting from residue 4 onwards (Fig. 4b). The sequence is markedly basic, reminiscent of the RNA-binding domains of *T*=3 plant viruses^41^ (Supplementary Fig. 4). Proceeding from residue 64 to residue 4, the polypeptide leaves the VP3 eight-stranded antiparallel β-barrel and passes along close to a five-fold axis to insert into a symmetry-related VP3 on the inner surface of the protein shell (Fig. 4). There are five prominent, ∼5-nm-long spiralling regions of density extending radially from each five-fold axis at the inner surface of the capsid (Fig. 4). There is a considerable amount of density in addition to that corresponding to the residues of the VP3 N-terminal extension, which we identify as RNA in part based on the shape of the electron density (Fig. 4a; Supplementary Fig. 6). We note that a feature at a similar location was also observed in human parechovirus, although at lower resolution, such that it was not possible to identify the nature of the protein–RNA interaction^15^. Electrostatic interactions dominate the contact between the VP3 N terminus and the RNA stem–loop (Fig. 4c). The N-terminal polypeptide (residues 1–64) comprises 13 basic resides, most of which are clustered within the first 20 residues. A total of 65 basic residues from mainly the N-terminal segments of the VP3 subunits around the five-fold axes, but also including a region of VP1, produce a positively charged environment that partly counteracts the abundant negative charge of the RNA phosphate groups. Residues including Arg 6, Lys 7, Lys 9, Lys 12, Lys 14, Arg 49, Arg 62, Lys 64 of VP3 and Arg 225 of VP1 are engaged in interactions with the ordered RNA (Fig. 4b); of these, most of the VP3 residues are conserved across the genus, while residue 225 of VP1 is strictly conserved.

Our interpretation of the RNA density places 15 bases of RNA in close association with the N terminus of VP3, although the resolution is insufficient to identify them individually. The strength of the icosahedrally averaged density implies that the RNA is essentially fully occupied (Fig. 4a; Supplementary Fig. 6). Throughout the capsid the ordered RNA comprises some 900 bases, 12% of the viral genome. We note that Seal *et al*.^42^ observed that HPeV-1 contains a large (40%) fraction of hairpin RNA compared with PV RNA and that modelling of the genomic 5′ end showed unique secondary structure folding of this region^25^; we propose that this structural constraint is a common feature of parechoviruses.

### Evolution and implications for assembly

**Figure 5:**
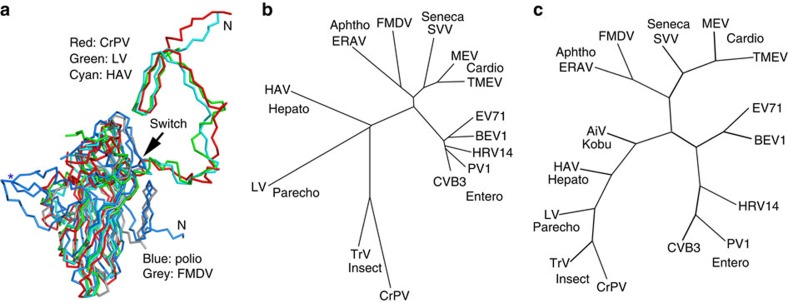
Phylogeny. (**a**) Superposition of LV VP0 with VP2 of poliovirus, FMDV, HAV and CrPV. The N terminus of LV VP0 folds similarly to that of HAV and CrPV but differently to other picornaviruses. The star marks the EF loop that forms part of the south wall of the canyon in poliovirus. (**b**) Structure and (**c**) sequence-based phylogenetic trees of representative picornaviruses and cripaviruses, respectively: CVB3, coxsackievirus B3; PV1, poliovirus type 1; HRV14, human rhinovirus 14; BEV1, bovine enterovirus type 1; EV71, EV-A71; TMEV, Theilers virus; MEV, Mengo virus; SVV, Seneca valley virus; FMDV, foot-and-mouth disease virus; ERAV, equine rhinitis A virus; HAV, hepatitis A virus; AiV, Aichi virus^59^; TrV, triatoma virus; CrPV, cricket paralysis virus.

Overall, the structure of LV is most similar to that of HAV (Fig. 5). Furthermore, LV possesses a number of structural features also observed in insect viruses, including the VP2 N-terminal domain swap (Fig. 5a), the presence of a basic VP3 N-terminal ‘arm'^43^ and the observation of well-ordered genome RNA with numerous protein/RNA interactions^44^. Indeed, both structure and sequence-based phylogeny suggests that LV is the closest picornavirus to the insect picorna-like viruses (Fig. 5b,c).

## Discussion

One of the key distinguishing features of LV is the long extension at the C terminus of VP1. Other picornaviruses such as CV-A9 and the closely related HPeV also have VP1 C-terminal extensions, although shorter, and these function in cell attachment^15^. This is the most likely role of this extension, presumably giving the virus scope to attach to a different receptor, although a role in particle assembly is also possible as in HAV^21^. In line with this, mutations that increase replication efficiency map to this region or the adjacent VP0 EF loop. Increased replication efficiency was also associated with induction of an apoptotic response in GMK cells, as indicated by detection of activated caspase-3 and DNA fragmentation^40^. It is conceivable that this extension, with a rich collection of basic residues might also possess an alternative function in regulating host gene expression.

The observation of 15 bases of relatively well-ordered RNA associated with the N termini of VP3 near the five-fold axes is also significant in terms of the *Picornaviridae* family. Well-ordered genome RNA has thus far only been observed in parechoviruses^15^. For other genera of the family, only occasional isolated bases have been observed. This suggests that members of the Parechovirus genus may use a mechanism of passive genome encapsidation whereby VP3 acts as an RNA chaperone, organizing the genome into a condensed structure compatible with the icosahedral symmetry of the particle through numerous largely electrostatic interactions with short RNA segments with preformed stem–loop structures. Such a mechanism would be significantly different from the model for enterovirus packaging, which involves interaction between capsid proteins and non-structural proteins in the RNA replication complex, as described for PV^7^, but is not inconsistent with the observation of RNA secondary structures described as packaging signals in Aichi virus^8^ (genus *Kobuvirus*, see Fig. 5c) and the icosahedral RNA plant virus, satellite tobacco necrosis virus^4^. This may also be relevant to the reported lack of VP0 cleavage in parechoviruses, since this maturation cleavage is thought to happen autocatalytically in the presence of RNA, and may be a final quality assurance step required, for example, by genera such as enteroviruses. Substantial portions of ordered RNA have been reported in insect, plant, other animal viruses and bacteriophages^45^,^46^,^47^,^48^, and basic regions, often located at the N- or C termini of the coat proteins, are found in several families of icosahedral viruses where they are thought to be involved in RNA–protein interactions responsible for encapsidating the viral genome^1^. Similar to some insect viruses, LV bears such a basic A/LRM motif at the VP3 N terminus, which is seen to bind viral RNA. This binding would not seem to be directly sequence dependent, but governed by electrostatic effects in conjunction with local RNA structure. A role for the highly basic 20 N-terminal residues of LV VP3, in association with RNA, in directing the assembly and dimensioning of the pseudo *T*=3 particle^49^, would be consistent with the lack of observation of empty parechovirus particles in the viral life cycle. In the absence of the RNA the charge on the individual protomers may be repulsive, perhaps blocking the assembly of the pentamer^4^. It has been shown for nodaviruses^47^,^50^ that selection of non-viral RNA for packaging results in the formation of more dynamic particles. It may be that more recently evolved picornaviruses use more specific encapsidation signals in the RNA^51^ rather than an electrostatic effect. Indeed, other evidence presented here such as the VP2 domain swap and absence of a VP1 pocket suggests that LV, similar to HAV, retains a number of structural and functional features characteristic of primordial picornaviruses, which may have had their origins in insect viruses. Understanding the physical characteristics of LV and its assembly should help in devising methods to prevent and alleviate the infection and spread of this virus.

## Methods

### Infectious cDNA *in vitro* transcription and transfection

An infectious cDNA clone, pLV87-012G, containing the full genome sequence of LV-1 87-012G, was linearized using the NotI site encoded downstream of the poly-A tail and transcribed *in vitro* using a MEGAscript T7 Transcription kit (Ambion). RNA was transfected into subconfluent Vero cells using Escort IV reagent (Sigma). CPE was initially weak and increased CPE was observed over five passages following which the cell lysates were used to infect GMK cells.

### Virus production and purification

LV was grown in 28 × 175-cm^2^ flasks of GMK cells infected at a multiplicity of infection of 2. The cells were cultured in DMEM (Sigma) supplemented with 1% fetal bovine serum (Gibco) until 90% of cells exhibited a CPE. Both cells and virus-containing supernatant were collected, frozen and thawed three times and then centrifuged to remove cell debris. The supernatants were centrifuged for 3 h at 95,000*g* and viral pellets resuspended in PBS buffer+0.01% Triton X-100, then loaded onto 15–45% (w/v) sucrose density gradients and centrifuged at 222,000*g* max for 1.5 h in an SW41 rotor at 4 °C. Further details have been described previously^12^.

### Negative-stain electron microscopy

Aliquots of purified samples were diluted to 0.2 mg ml^−1^ and deposited onto glow-discharged, Formvar/carbon-coated copper grids (Electron Microscopy Sciences, Hatfield, USA). After 30 s incubation, the excess sample was blotted away and the grids washed twice with deionized water. Samples were stained with 1% (w/v) uranyl acetate for 45 s and excess stain was removed by blotting. Grids were examined on a Tecnai T12 (FEI, Hillsboro, OR) transmission electron microscope operated at 80 kV. Images were acquired on a 4 × 4-k-High-sensitivity FEI Eagle camera (FEI) at a nominal magnification of × 67,000.

### Cryo-EM and data collection

A 3-μl aliquot of purified LV virions (∼1 mg ml^−1^) was applied to a freshly glow-discharged 400-mesh holey carbon-coated copper grid (C-flat, CF-2/1-2C; Protochips Inc.) and blotted for 3 s, in 70% relative humidity for plunge-freezing (Vitrobot; FEI) in liquid ethane. Cryo-EM data were collected using an FEI Tecnai G2 Polara microscope (FEI) operated at 300 kV, equipped with an energy filter (GIF Quantum; Gatan, Pleasanton, CA) operating in zero-loss mode (20 eV energy selecting slit width) and a direct electron detector (K2 Summit; Gatan). Micrographs were collected with a defocus between 1.5 and 3 μm as movies (25 frames, each 0.2 s) in single electron counting mode using SerialEM^52^ at a calibrated magnification of × 37,027, resulting in a pixel size of 1.35 Å.

### Image processing and 3D reconstruction

The micrographs were corrected for beam-induced drift by aligning and averaging the individual frames of each movie using MOTIONCORR^53^,^54^. Particles were selected automatically using ETHAN^54^ and then manually screened with the boxer program in EMAN^55^. Contrast transfer function (CTF) parameters for drift corrected micrographs were estimated using a GPU accelerated program Gctf (Kai Zhang, Gctf: real-time CTF determination and correction, 2015, http://www.biorxiv.org/content/early/2015/07/12/022376). Micrographs showing signs of astigmatism or significant drift were discarded and not used for further analysis. A total of 5,558 particles from 288 micrographs were subjected to 2D alignment and 3D reconstruction using Relion 1.3 (ref. 28) by following recommended gold standard refinement procedures^28^ and applying icosahedral symmetry. Two initial models for LV were generated, one was created by EMAN2 (ref. 27); another was generated by filtering the reconstructed HAV structure to 30 Å; the independent initial models all converged to the same result. Several cycles of 2D classification and 3D refinement were used to further select the particles for final refinement. The final resolution was evaluated using the ‘gold' standard Fourier shell correction (threshold=0.143 criterion)^30^,^31^ of the two truly independent reconstructions (Supplementary Fig. 2). The data set and refinement statistics are summarized in Table 1.

### Model building and refinement

The atomic models of LV VP0, VP1 and VP3 were built *de novo* into density with the structures of HAV capsid proteins as a guide, using COOT^56^. REFMAC^57^ was used to calculate the difference map that highlighted the areas where the model was incorrect. Models were further improved by iterative positional and B-factor refinement in real space using Phenix^32^ and rebuilding in COOT^56^. Only coordinates were refined, the maps were kept constant. Each round of model optimization was guided by cross-correlation between the map and the model. Refinement statistics are given in Table 2 as evaluated by Molprobity^58^ functions integrated in Phenix.

### Thermal stability assay

PaSTRy^26^ experiments were performed with an MX3005p RT–PCR instrument (Agilent) using both SYTO9 and SYPRO-Red (Invitrogen) dyes to detect the presence of RNA and the exposed hydrophobic regions of proteins, respectively. Fifty-microlitre reactions were set up in a thin-walled PCR plate (Agilent), containing 1 μg of the LV virus, 5 mM SYTO9 and 3 × SYPRO-Red in PBS (pH 7.4), and the temperature ramped from 25 to 99 °C. Fluorescence was recorded in triplicate at 1-°C intervals.

## Additional information

**Accession codes:** Coordinates are deposited in the Protein Data Bank under accession code 3JB4. Cryo-EM reconstructions are deposited in the EM Data Bank under accession codes EMD-6394 (3.78 Å) and EMD-6395 (4.3 Å).

**How to cite this article:** Zhu, L. *et al*. Structure of Ljungan virus provides insight into genome packaging of this picornavirus. *Nat. Commun.* 6:8316 doi: 10.1038/ncomms9316 (2015).

## Supplementary Material

Supplementary Information Supplementary Figures 1-6 and Supplementary References

## Figures and Tables

**Table 1:** Cryo-EM imaging and data processing statistics.

Micrographs (total) 292; micrographs (used) 288	
Particles selected	5,867
Particles included in final reconstruction	5,558
Sampling, Å per pixel	1.35
Defocus range, μm	1.5–3.0
Resolution, Å (gold standard FSC=0.143 criterion)	3.78

FSC, Fourier shell correlation.

**Table 2:** Model refinement statistics.

R.m.s.d. (bond angles, °)	1.573
R.m.s.d. (bond lengths, Å)	0.010
Ramachandran statistics	
Most favoured (%)	90.7
Allowed	8.2
Outliers	1.1
*R*_work_/*R*_free_ (%)	29.7/31.3

r.m.s.d., root mean squared deviation.
