# Supplementary material for: Structure of Ljungan virus provides insight into genome packaging of this picornavirus
Source: Nat Commun. 2015 Oct 8;6:8316. doi: 10.1038/ncomms9316 (PMC4633645; doi:10.1038/ncomms9316)
Supplement: Supplementary Information — Supplementary Figures 1-6 and Supplementary References [file ncomms9316-s1.pdf]

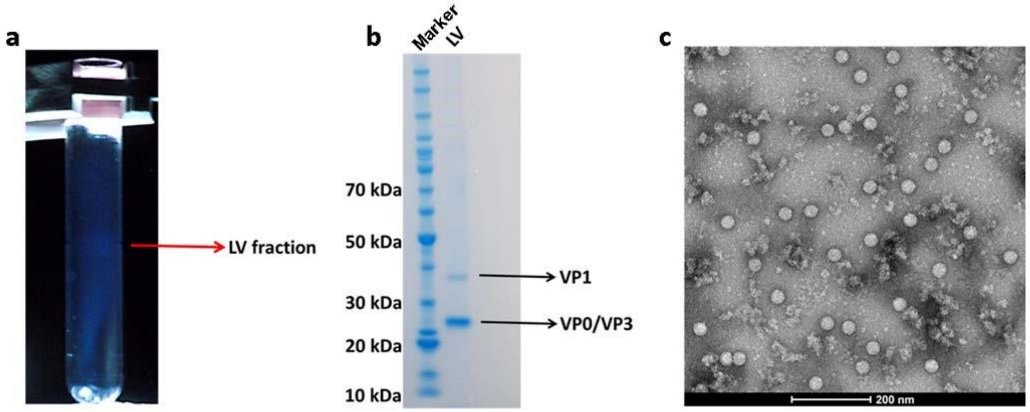


**Supplementary Figure 1 | LV purification and characterization. (a)** Zonal ultracentrifugation of a 15% to 40% sucrose density gradient for the purification of LV, as described in Methods. Only one predominant particle type was observed and the λ260/λ280 absorbance ratio was 1.66, indicating that the LV particles contain the RNA genome. **(b)** SDS-PAGE for viral protein composition analysis using a NuPAGE 4-12% Bis-Tris Gel (Invitrogen). The calculated molecular weights of VP0, VP1 and VP3 were 28.2 kDa, 27.7 kDa, 33.1 kDa respectively. **(c)** Negative stain electron microscopy of LV particles.

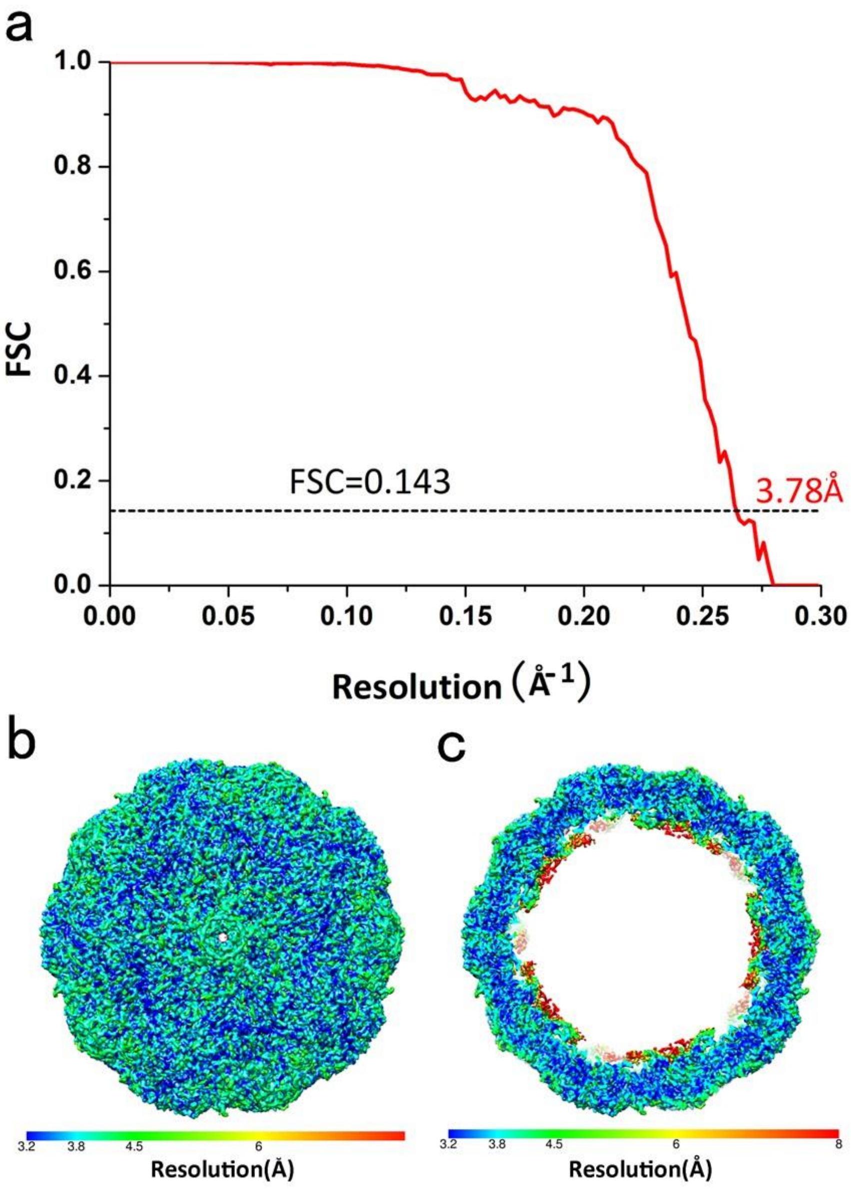


**Supplementary Figure 2 | Resolution evaluation of Cryo-EM map of LV. (a)** The gold-standard FSC curves of the final maps. The resolution at FSC=0.143 is 3.78 Å. **(b) and (c)** The final LV map was analyzed by ResMap[1] showing a resolution distribution from 3.2 to 8Å. Core parts of the map have resolution of ~3.2Å, the resolution of the interior parts of the map (corresponding to RNA density) is ~7-8 Å.

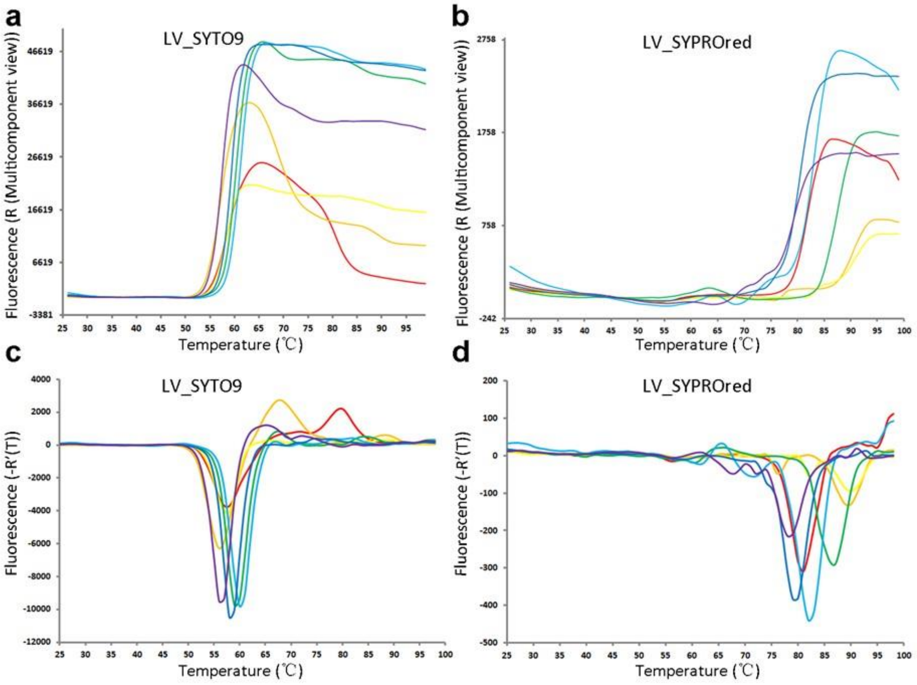


**Supplementary Figure 3 | Thermal stability of LV particles**. The stability of LV particles across the pH range from 4.0 to 10.0 was characterized by differential scanning fluorimetry assays using dyes SYTO9 and SYPRO RED to detect RNA exposure and protein melting, respectively. (**a**) The raw fluorescence traces of LV particles incubated with SYTO9 and (**b**) with SYPROred; their first derivatives are shown in (**c**) and (**d**), respectively. The colour scheme is red (pH 4.0), orange (pH 5.0), yellow (pH 6.0), green (pH 7.0), sky blue (pH 8.0), blue (pH 9.0) and purple (pH 10.0).

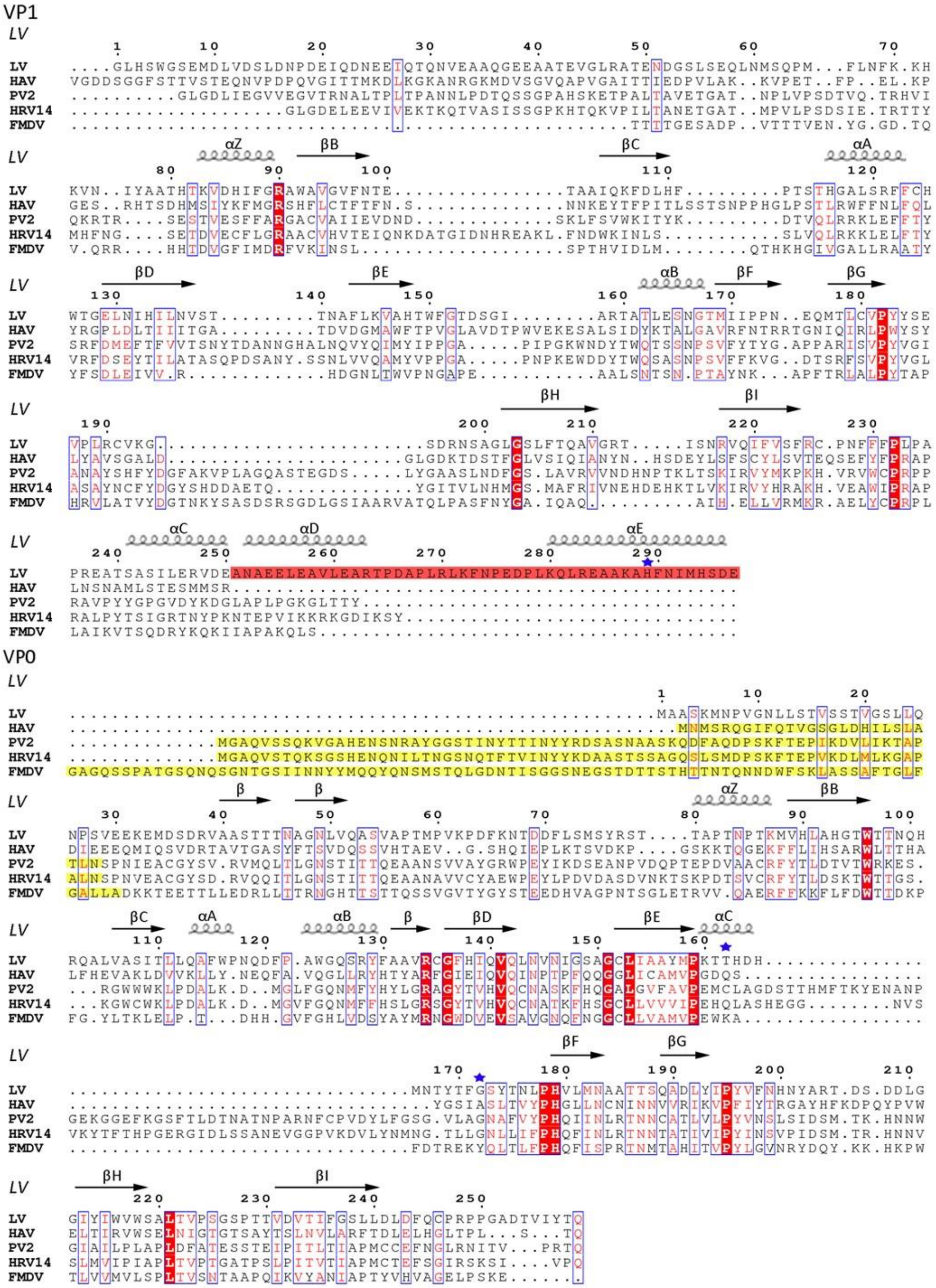
VP1
VP0
LV
HAV
PV2
HRV14
FMDV

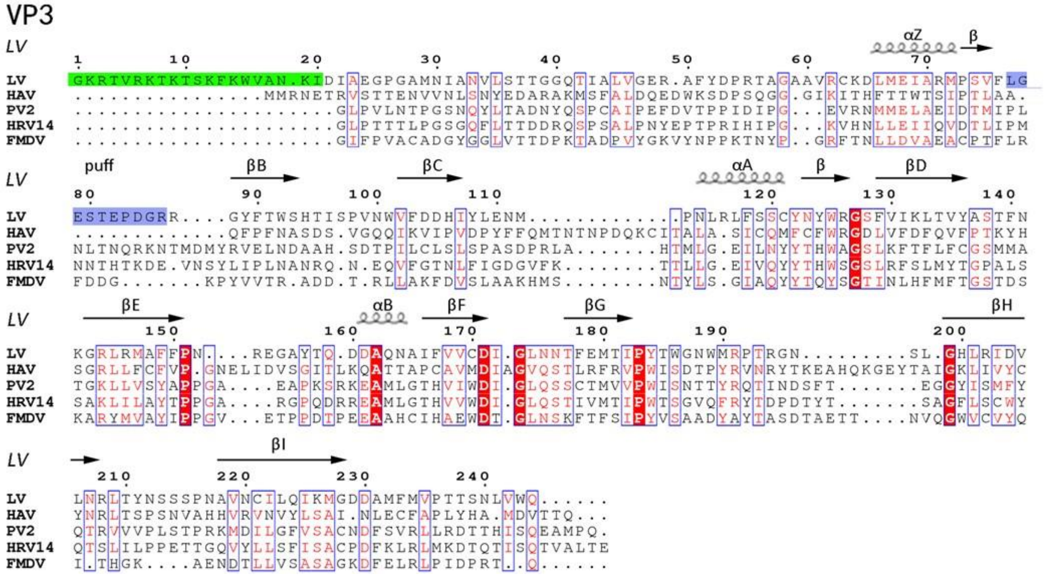


**Supplementary Figure 4 | Structure-based sequence alignment.** Espript[2] representation of a structure-based sequence alignment of VP1, VP0 and VP3 of the LV particle with representative piconaviruses, including hepatitis A virus (HAV), poliovirus type 2 (PV2), human rhinovirus 14 (HRV14) and foot-and-mouth disease virus (FMDV) (PDB codes: 4QPI, 1EAH[3], 4RHV[4] and 1ZBA[5] respectively). The secondary structural elements for LV are shown, also above the sequences are symbols which show features described in the text. The unique extension of VP1 C-terminus in LV is highlighted in red. An approximately 20 amino acids extension enriched with basic residues to the N terminus of VP3 in parechovirus is highlighted in green. VP4 sequences of HAV, PV2, HRV14 and FMDV are shown in yellow. A puff configuration in VP3 of LV is represented in blue. The blue stars represent the cell adapted mutations. The residue numbers are for LV.

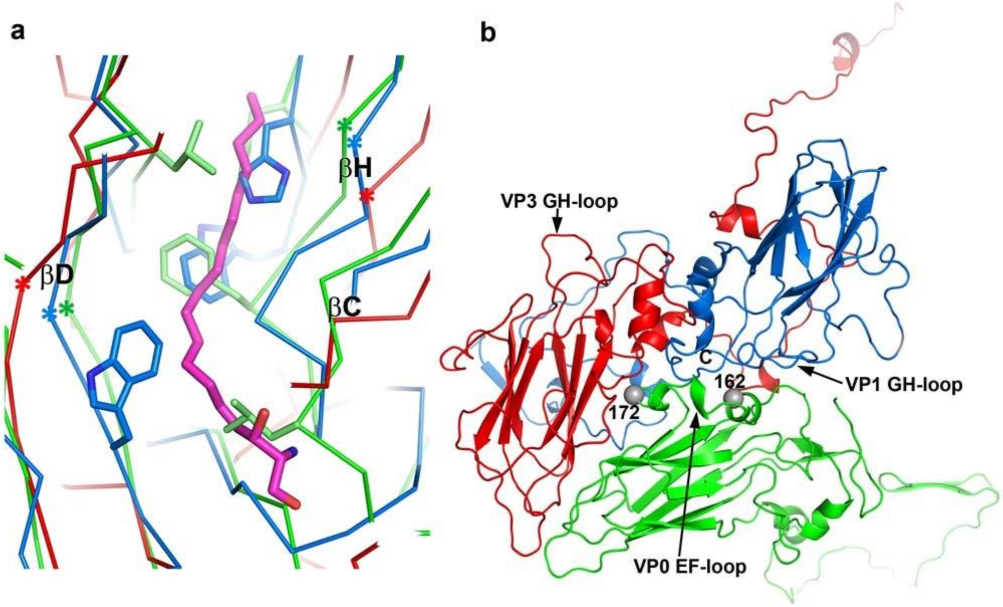


**Supplementary Figure 5 | Pocket analysis and cell adapted mutations**. (**a**) Close-up view of LV (blue) and HAV (green) compared with VP1 pocket factor binding region of EV71 (red; pocket factor, magenta), note the shift of strand C and strand H and occlusion of pocket entrance. Bulky side chains occlude the HAV and LV pocket. (**b**) Ribbon diagram of LV protomer with cell adapted mutations (residues 162 and 172) in VP0 EF-loop shown as grey spheres. The third mutation Y289H at VP1 C-terminal region is not built in the model due to the lower resolution electron density map. VP1, VP0 and VP3 are coloured in blue, green and red, respectively.

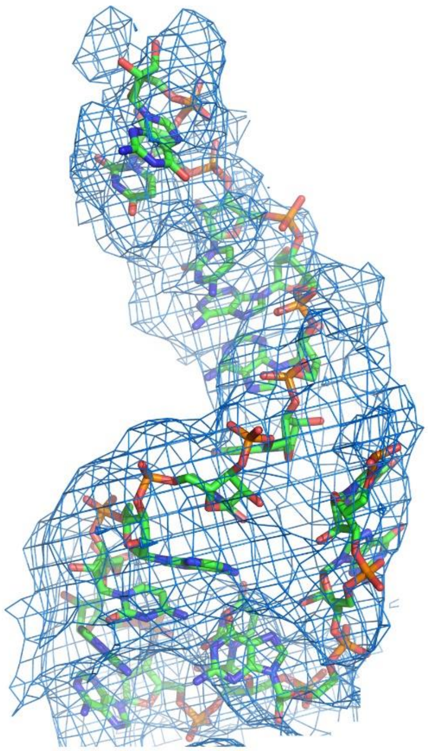

**Supplementary Figure 6 | Close-up view of electron density for the ordered RNA.** The map is contoured at 3σ and the resolution is 7-8 Å (Supplementary Fig. 2). The nucleotide sequence is arbitary.

**Supplementary References**


1. Kucukelbir, A., Sigworth, F.J. & Tagare, H.D. Quantifying the local resolution of cryo-EM density maps. *Nat Methods* **11**, 63-5 (2014).
2. Gouet, P., Courcelle, E., Stuart, D.I. & Metoz, F. ESPript: analysis of multiple sequence alignments in PostScript. *Bioinformatics* **15**, 305-8 (1999).
3. Lentz, K.N. et al. Structure of poliovirus type 2 Lansing complexed with antiviral agent SCH48973: comparison of the structural and biological properties of three poliovirus serotypes. *Structure* **5**, 961-78 (1997).
4. Arnold, E. & Rossmann, M.G. The Use of Molecular-Replacement Phases for the Refinement of the Human Rhinovirus-14 Structure. *Acta Crystallographica Section A* **44**, 270-282 (1988).
5. Fry, E.E. et al. Structure of Foot-and-mouth disease virus serotype A10 61 alone and complexed with oligosaccharide receptor: receptor conservation in the face of antigenic variation. *J Gen Virol* **86**, 1909-20 (2005).
